# 

**Published:** 1860-10

**Authors:** 


### CONTENTS FOR OCTOBER, 1860.

**ORIGINAL COMMUNICATIONS.**

Report on the Changes in the Composition and Properties of the Milk in the Human Female, produced by Menstruation and Pregnancy; also, on the Food most Proper for Infants when Deprived of the Milk of the Mother. By N. S. DAVIS, M.D... 577
Hydrocyanic Acid should be Stricken from the List of Officinal Preparations. (From a Paper on Hydrocyanic Acid, by Dr. F. MAHLA.)........ 589
Osteöid Cancer of the Femur........ 592
Stramonium in Neuralgia. By A. YOUNG, M.D., of Prescott, Wis........ 594
Proceedings of the Medical Society of the City of Galesburg, Held Sept. 7th, 1860...... 595
Clinical Cases. By N. S. DAVIS, M.D., &c........ 597

**BOOK AND PAMPHLET NOTICES.**

The Principles and Practice of Modern Surgery. By ROBERT DRUITT........ 600
On the Theory and Practice of Midwifery. By FLEETWOOD CHURCHILL, M.D., &c. With Additions by D. FRANCIS CONDIE, M.D., &c ........ 601
O'REILLEY on the Placenta and Nervous System........ 602

**SELECTIONS.**

Infantile Pathology and Therapeutics. By A. JACBI, M.D........ 603
Illustrations of Hospital Practice. Pennsylvania Hospital.—Service of Dr. MEIGS.... 608
Obstetrics.—Iodine Injections in Ovarian Cysts. By Prof. SCUH........ 611
Practice of Medicine.—On the Rational Treatment of Delirium Tremens. By Professor DUNGLISON. In a Letter to Professor LAYCOCK........ 614
Lecture on Parturient Hemorrhage. By T. GILLARD THOMAS, M. D........ 617

**EDITORIAL.**

Legislation on Medical Subjects........ 528
Medical Department of Lind University. Chicago City Dispensary........ 629
Mercy Hospital Report........ 630
Puffing........ 631
New York Medical College and Charity Hospital........ 632
Medical Department of University of Michigan. Epilepsy........ 633
Physiology and Pathology of the Spleen. Medical College of Ohio........ 634

### MEDICAL DEPARTMENT
OF
### LIND UNIVERSITY.

